# Venomics of the Anchor Coral Snake *Micrurus ancoralis*: Composition, Toxicological Profile, and Neutralization by Commercial Antivenom

**DOI:** 10.3390/tropicalmed11080223

**Published:** 2026-08-10

**Authors:** Paola Rey-Suárez, Jonard David Echavarría-Rentería, Jeisson Gómez-Robles, Jaime Andrés Pereañez, Mónica Saldarriaga-Córdoba, Bruno Lomonte, Julián Fernández, Vitelbina Núñez

**Affiliations:** 1 Facultad de Ciencias Exactas y Aplicadas, Institución Universitaria ITM, Medellín 050034, Colombia; 2 Facultad de Ciencias Naturales y Exactas, Universidad Tecnológica del Chocó, Quibdó 270001, Colombia; jd07echavarria@hotmail.com; 3 Grupo de Investigación en Toxinología, Alternativas Terapéuticas y Alimentarias, Universidad de Antioquia, Medellín 050010, Colombia; jeisson.gomez1@udea.edu.co (J.G.-R.); jaime.pereanez@udea.edu.co (J.A.P.); vitelbina.nunez@udea.edu.co (V.N.); 4 Centro de Investigación en Recursos Naturales y Sustentabilidad (CIRENYS) and Escuela de Medicina Veterinaria, Universidad Bernardo O’Higgins, Santiago 8320000, Chile; 5 Instituto Clodomiro Picado, Facultad de Microbiología, Universidad de Costa Rica, San José 11501, Costa Rica; bruno.lomonte@ucr.ac.cr (B.L.); 6 Escuela de Microbiología, Universidad de Antioquia, Medellín 050010, Colombia

**Keywords:** *Micrurus ancoralis*, coral snake, anticoral antivenom, venomics, immunorecognition, Colombia

## Abstract

Coral snakes of the American continent are classified into two genera, *Micruroides* and *Micrurus*, with as many as 91 species recognized in the latter. Although envenomings caused by *Micrurus* are rare, they can be life-threatening due to neurotoxic effects leading to flaccid paralysis and potential respiratory failure. *Micrurus ancoralis* is found in the Pacific lowlands and along the western slope of the cordillera occidental Cordillera in Colombia. Although this species is relatively common within its distribution range, its phylogenetic position and the characterization of its venom have not been investigated. The results of phylogenetic analysis placed *M. ancoralis* within the triadal/bicolor clade of species with the characteristic triad pattern and group bicolor. Proteomic characterization of the venom showed predominance of phospholipase A_2_ (PLA_2_) enzymes in its composition, with 51.7% of the total protein content, followed by three-finger toxins (3FTxs; 23.2%) and lower proportions of proteins belonging to several other minor families. Out of 28 chromatographic venom fractions obtained, the seven most abundant were evaluated for toxicity, with three of them (F7, identified as a 3FTx, and F18 and F20 (both identified as PLA_2_s)) showing lethal activity by the intraperitoneal route in mice. The PLA_2_ activity of F20 was confirmed and its edema-inducing, myotoxic, and lethal effects in mice were demonstrated. A therapeutic equine anti-coral antivenom (INS, Colombia) against coral snake envenomings immunorecognized the whole venom and its fractions in ELISA tests and was able to neutralize 2 × LD_50_ (medial lethal dose) of *M. ancoralis* venom by preincubation, with an estimated potency of at least 0.2 mg venom/mL antivenom. This result suggests that envenomings by *M. ancoralis* could be effectively treated by this antivenom.

## 1. Introduction

In the Americas, coral snakes are the only representatives of the Elapidae family. They are classified into two genera: *Micruroides* and *Micrurus* [1]. The first has a single species, *M. euryxanthus*, while the second includes 91 recognized species [2]. Coral snakes are widely distributed across the Americas, ranging from southern United States to Argentina [3,4]. Human envenomings caused by these species are estimated at about 3% of all snakebites, and considered life-threatening due to the neurotoxic action of their venoms [5,6,7]. The neurotoxic effects often manifest initially by bilateral palpebral ptosis and may progress to a flaccid diaphragm paralysis, leading to respiratory failure and death in severe cases. These effects are induced by three-finger toxins (3FTxs) and group IA phospholipases A_2_ (PLA_2_s). 3FTxs block the binding of acetylcholine (ACh) to its receptors in the neuromuscular junction [8], whereas PLA_2_s hydrolyze glycerophospholipids on the presynaptic membrane, leading to an irreversible inhibition of ACh release [9].

**Figure 1:**
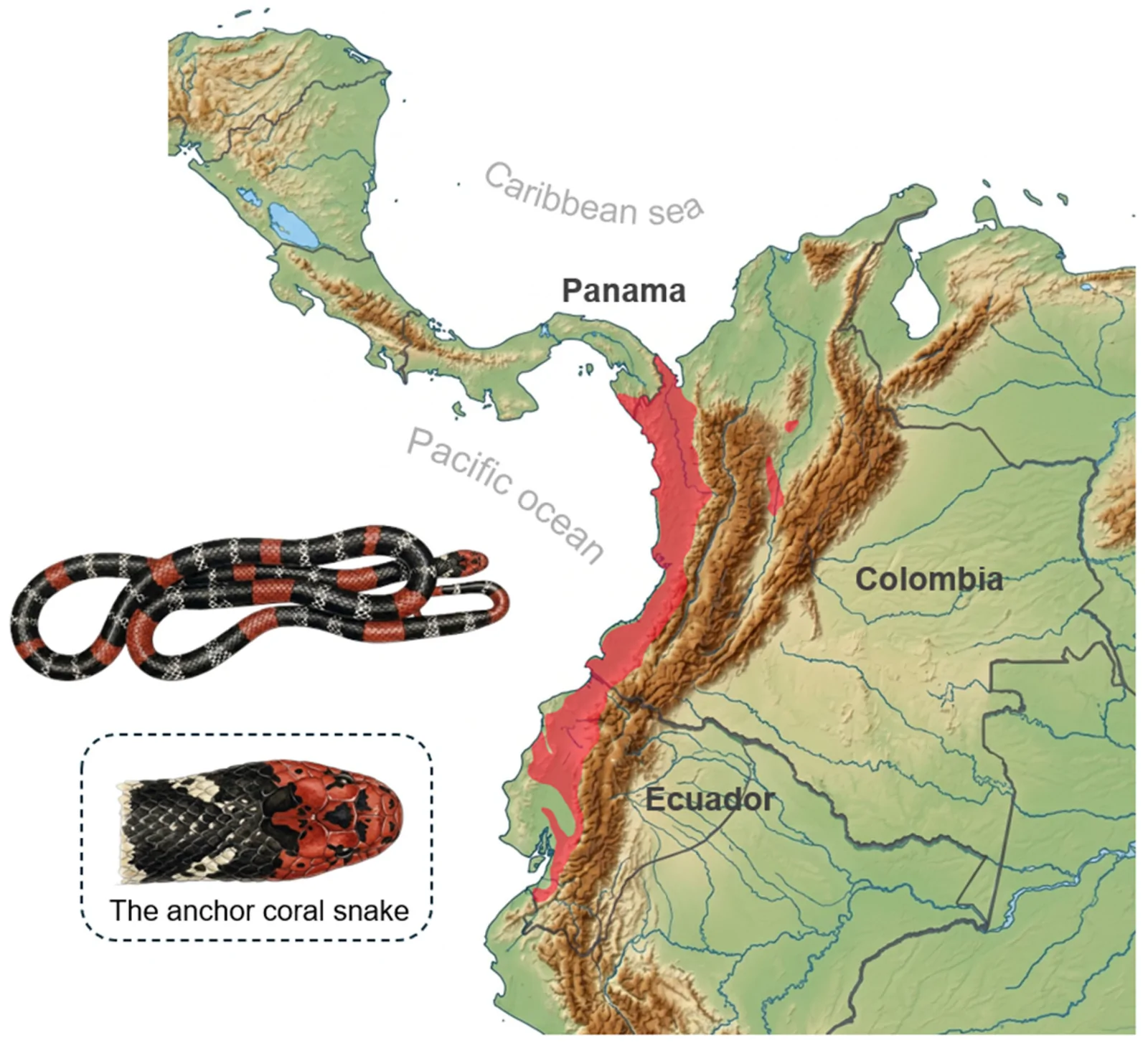
Geographic distribution of *Micrurus ancoralis* in the Americas. General illustration of the specimen along with a dotted inset highlighting the distinctive cephalic markings; these anchor-shaped patterns give rise to the common name “anchor coral snake” (illustrations were generated by Gemini AI tool Version 3.5 Flash; Google (Mountain View, CA, USA), 2026, based on original photographs provided by the authors). The red area represents the geographic distribution of the species.

*Micrurus ancoralis*, commonly known as the anchor coral snake due to its distinctive anchor-shaped nuchal band (Figure 1), belongs to the South American triadal group and is one of the largest coral snake species, reaching lengths of up to 150 cm [10]. This species is distributed from southeastern Panama to the western slopes of the Andes in southwestern Ecuador [11]. It inhabits tropical rainforests and low mountain wet forests, ranging from near sea level to approximately 1500 m above sea level [10,12]. In Colombia, *M. ancoralis* is found in the Pacific lowlands and along the western slope of the Western Cordillera, spanning the departments of Antioquia, Cauca, Chocó, Nariño, Risaralda, and Valle del Cauca (Figure 1) [11,13]. Recently, through the validation of citizen science data, its distribution has been extended to include the tropical forests of the eastern slope of the Central Cordillera in the Magdalena River Valley [14].

Although *M. ancoralis* is relatively common within its distribution range, its venom has not been previously analyzed, and no information is available on its enzymatic and toxic effects. In this study, we conducted a proteomic and functional characterization of this venom from specimens collected in Colombia, along with an evaluation of the ability of a therapeutic equine anti-coral antivenom to immunorecognize and neutralize this venom. In addition, a molecular phylogenetic reconstruction was performed using the ND4 gene aiming to determine the position of *M. ancoralis* within a tree of *Micrurus* species.

## 2. Materials and Methods

### 2.1. Venom and Antivenom

Venom was manually extracted from four adult male and female specimens of *Micrurus ancoralis* kept in captivity at the Serpentarium of the University of Antioquia from Quibdó-Chocó (Colombia). For biological activity assays, proteomic profiling, toxicity and neutralization evaluations, a pooled venom sample derived from these individuals was utilized. Individual venom extractions from three male specimens were analyzed independently to assess intraspecies variation. The venoms were lyophilized and stored at −20 °C until use. The genetic resource access permits covering these individuals were: RGE: 0156–9 and RGE: 0156–15. Neutralization assays were conducted using a commercially available equine anticoral antivenom produced by Instituto Nacional de Salud from Bogotá, Colombia (INS; Batch No. 19AMP01; Expiry date: November 2025). According to the manufacturer, the antivenom is prepared using a mixture of the venoms of *M. dumerilii*, *M. mipartitus*, *M. isozonus*, and *M. surinamensis* for immunization.

### 2.2. Reverse-Phase HPLC and SDS-PAGE Venom Fractionation

A 2 mg aliquot of *M. ancoralis* venom was reconstituted in 200 µL of 0.1% trifluoroacetic acid (TFA; Solution A) and centrifuged at 1250× *g* for 5 min to remove insoluble particulate matter. The supernatant was fractionated using a C_18_ reverse-phase column (Pinnacle, 250 × 4.6 mm, 5 µm particle size, 140 Å pore size; Restek, Bellefonte, PA, USA) coupled to a Shimadzu Prominence-20A chromatographic system (Shimadzu Corp., Kyoto, Japan), with spectrophotometric monitoring at 215 nm. Elution was performed at a constant flow rate of 1 mL/min utilizing a multi-step linear gradient toward Solution B (acetonitrile containing 0.1% TFA) as follows: an isocratic step at 5% B for 5 min, followed by 5–15% B over 10 min, 15–45% B over 60 min, and 45–70% B over 12 min [15]. Chromatographic fractions were manually collected based on absorbance peaks at 215 nm, evaporated to dryness using a Vacufuge Plus vacuum concentrator (Eppendorf, Hamburg, Germany), and reconstituted in deionized water. Protein concentration was quantified by measuring absorbance at 280 nm using a NanoDrop 2000 spectrophotometer (Thermo Fisher Scientific, Waltham, MA, USA), assuming an extinction coefficient where an absorbance of 1.0 corresponds to a 1 mg/mL protein solution. Following concentration normalization, fractions were thermally denatured at 95 °C for 5 min under reducing conditions using a sample buffer supplemented with 2-mercaptoethanol and resolved via SDS-PAGE on 12% polyacrylamide gels alongside molecular weight markers. Protein bands were visualized through staining with Coomassie Brilliant Blue R-250.

### 2.3. Proteomic Characterization of Venom: ‘Venomics’ Strategy

SDS-PAGE bands from each Reverse-phase HPLC fraction were excised and subjected to reduction with dithiothreitol (10 mM) and alkylation with iodoacetamide (50 mM), followed by incubation with sequencing-grade trypsin. An automated workstation (Intavis, Germany) was used, following the manufacturer’s instructions. A nano-Easy 1200 chromatograph in-line with a Q-Exactive Plus mass spectrometer (Thermo Fisher) was used for nESI-MS/MS analysis of resulting peptides. A C_18_ trap column (75 μm  ×  2 cm, 3 μm particle size, Acclaim PepMap 100, Thermo Scientific) was loaded with five μL of each peptide digest and after a wash with 0.1% formic acid (solution A), peptides were separated through a C_18_ Easy-spray column (15 cm  ×  75 μm, 3 μm particle size) using a flow rate of 200 nL/min. Peptides were eluted over 45 min using a gradient of solution B (80% acetonitrile, 0.1% formic acid) as follows: 1–5% B in 1 min, 5–25% B in 30 min, 25–79% B in 6 min, 79–99% B in 2 min, 99% B for 6 min. The mass spectra were acquired in positive mode at 1.9 kV, capillary temperature of 230 °C, using 1 μscan in the range 400–1600 *m*/*z*, maximum injection time of 100 ms, AGC target of 3  ×  10^6^, and a resolution of 70,000. The top 10 ions with 2–5 positive charges were fragmented with an AGC target of 1  ×  10^5^, a maximum injection time of 110 ms, a dynamic exclusion time of 5 s, a resolution of 17,500, a normalized collision energy (NCE) of 28 and an isolation window of 1.4 *m*/*z*. The resulting MS/MS spectra were searched against protein sequences contained in the UniProt/SwissProt Serpentes database (https://www.uniprot.org), released and downloaded 1 March 2024, with 361,322 entries, using PEAKS X (Bioinformatics Solutions, Waterloo, ON, Canada). Matches were assigned to known protein families by similarity. A parent mass error tolerance of 15.0 ppm and a fragment mass error tolerance of 0.5 Da were used. Cysteine carbamidomethylation was set as a fixed modification, while deamidation of asparagine or glutamine, and methionine oxidation were set as variable modifications, allowing up to 3 missed cleavages by trypsin. Parameters for match acceptance were set to FDR  <  0.1% controlled at the peptide-spectrum match (PSM) level, detection of at least one unique peptide, and −10 lgP protein score ≥ 50. To determine relative abundances (percentage of the total venom proteins) of protein families, the ratio of the sum of the areas of the reverse-phase chromatographic peaks containing proteins from the same family to the total area of venom protein peaks in the reverse-phase chromatogram was used. When several SDS-PAGE protein bands were present in a reverse-phase fraction, densitometry was used to determine their proportions, with ImageLab v2.01 (Bio-Rad, Hercules, CA, USA). When a single SDS-PAGE band contained several proteins, the area under the curve of the peptide features found at the same *m*/*z* and retention times as the MS/MS scans were used to estimate proportions [15].

### 2.4. ‘Shotgun’ Proteomic Profiling

A total of 15 μg of venom was reduced in solution with 10 mM dithiothreitol for 30 min at 56 °C, followed by alkylation with 50 mM iodoacetamide for 20 min in the dark, and digestion with sequencing grade trypsin (37 °C overnight, enzyme-to-substrate *w*/*w* ratio of 1:50), in a total volume of 40 μL. After the addition of 0.5 μL of formic acid and concentration using a vacuum concentrator, peptides were redissolved in 40 μL of 0.1% formic acid (solution A), centrifuged (15,000× *g* for 5 min) and separated by RP-HPLC on a nano-Easy 1200 chromatograph (Thermo) in-line with a Q-Exactive Plus mass spectrometer (Thermo). A C_18_ trap column (75 μm × 2 cm, 3 μm particle, 100 Å; PepMap, Thermo) was loaded with 10 μL (3.7 μg) of peptide mixture and washed with 0.1% formic acid (solution A). Peptides were then separated at 200 nL/min on a C_18_ Easyspray column (75 μm × 15 cm, 3 μm particle; PepMap, Thermo). A gradient toward solution B (80% acetonitrile, 0.1% formic acid) with the following conditions was used: total time of 120 min (1–5% B in 1 min, 5–26% B in 84 min, 26–80% B in 30 min, 80–99% B in 1 min, and 99% B for 4 min) [16]. MS and MS/MS spectra were acquired and processed as described in the previous Section 2.3.

### 2.5. Functional Characterization of M. ancoralis Venom

#### 2.5.1. In Vitro Venom Activities

The synthetic monodisperse substrate 4-nitro-3-octanoyloxy-benzoic acid (4-NOBA) was employed to assess PLA_2_ activity. For each assay, 20 µg of venom, previously dissolved in 25 µL of buffer (10 mM Tris, 0.1 M NaCl, 10 mM CaCl_2_, pH 8.0), was combined with 200 µL of the same buffer and 25 µL of 4-NOBA (1 mg/mL in acetonitrile). Reaction mixtures were incubated for 60 min at 37 °C, following the protocol of Mora-Obando et al. [17], and product formation was quantified spectrophotometrically at 425 nm on a Nunc microplate reader. Negative controls consisted of wells containing all assay components except venom, and every sample was run in triplicate.

Azocasein hydrolysis was used to assess proteolytic activity. Venom (20 µg), dissolved in 20 µL of buffer (25 mM Tris, 0.15 M NaCl, 5 mM CaCl_2_, pH 7.4), was added to 100 µL of azocasein solution (10 mg/mL in the same buffer) and the mixture was left to react for 90 min at 37 °C. The reaction was stopped through the addition of 200 µL of 5% trichloroacetic acid; following centrifugation, a 100 µL aliquot of the resulting supernatant was mixed with an equal volume of 0.5 M NaOH, as described by Wang et al. [18], and absorbance was read at 450 nm. Venoms from *M. mipartitus*, *M. dumerilii*, and *Bothrops asper* were assayed in parallel for comparative purposes.

To determine L-amino acid oxidase activity, 20 µg of venom (dissolved in 10 µL of water) was added to 90 µL of a reaction mixture consisting of 250 µM L-leucine, 2 mM o-phenylenediamine, and 0.8 U/mL horseradish peroxidase in 50 mM Tris buffer (pH 8.0), with all reactions performed in triplicate on microplate wells. Following a 60-min incubation at 37 °C, 50 µL of 2 M H_2_SO_4_ was added to terminate the reaction, and absorbance was recorded at 492 nm according to the method of Kishimoto and Takahashi [19].

Amidolytic activity was assessed following the approach of Erlanger et al. [20], using BApNA (N-succinyl-arginine-p-nitroanilide; Sigma-Aldrich, Darmstadt, Germany) as substrate. The assay combined 50 µL of Tris-HCl buffer (10 mM Tris-HCl, 10 mM CaCl2, 100 mM NaCl, pH 8.0), 200 µL of 10 mM BApNA, and 10 µL of *M. ancoralis* venom (20 µg). Absorbance was measured at 415 nm after a 40-min incubation period at 37 °C.

#### 2.5.2. In Vivo Venom Activities

Experimental procedures involving live animals were carried out on Swiss-Webster mice of both sexes (18–20 g body weight), under the terms of license N°110 (2017) and License N°160 (2024), issued by the Institutional Committee for Care and Use of Laboratory Animals (CICUA) of the University of Antioquia.

To determine lethal potency, increasing doses of *M. ancoralis* venom, dissolved in 300 µL PBS, were administered intraperitoneally (i.p.) to groups of three mice, while a control group received PBS only. Mortality was monitored for up to 48 h post-injection, and the median lethal dose (LD_50_) was estimated using the Probit method [21].

To evaluate myotoxic potential, 5 µg of venom (in 100 µL PBS) was injected into the gastrocnemius muscle of groups of three mice, with control animals receiving an equivalent volume of PBS alone. Tail blood samples were collected 3 h post-injection, and plasma creatine kinase (CK) levels were quantified through a kinetic UV assay (Wiener Lab, CK-NAC UV-AA; Riobamba, Rosario, Argentina).

Edema-inducing capacity was assessed by injecting 5 µg of venom (diluted in 50 µL PBS) into the footpad of the right hind paw in three mice, with the contralateral control group receiving the same volume of PBS. Footpad swelling was quantified as a percentage increase relative to controls 2 h after injection [22].

#### 2.5.3. Toxicity Screening and Intact Masses of Venom RP-HPLC Fractions

To evaluate toxicity of the seven most abundant venom fractions, each one (50 μg in 300 μL PBS) was administered by i.p. route to two mice, and lethality recorded over 24 h. This screening corresponds to dose close to the estimated DL_50_ of the crude venom determined in this study. Additionally, the LD_50_ was determined for the most abundant fractions that caused lethality during screening, using doses between 20 and 80 μg (in 300 μL PBS) in groups of three mice. A control group received only PBS. The LD_50_ was calculated using Probits [21].

The intact mass of most abundant venom fractions was also determined via ESI-MS. To this end, the proteins were dissolved in a 50% acetonitrile/water mixture containing 0.1% formic acid (at a concentration of 50–100 μg/mL) and infused at 5 μL/min into a HESI source. A full MS scan was acquired over the *m*/*z* range of 800–2000, with a resolution of 140,000 (at *m*/*z* 240) in positive mode, using a Q-Exactive Plus instrument (Thermo). To determine monoisotopic masses, the obtained mass spectra—corresponding to multiply charged ion series—were deconvoluted using Freestyle v.1.5 software (Thermo) [23].

### 2.6. Immunorecognition and Neutralization of M. ancoralis Venom by the INS Anticoral Antivenom

The immunoreactivity of the Instituto Nacional de Salud (INS) equine anticoral antivenom against whole *M. ancoralis* venom was quantified via an enzyme-linked immunosorbent assay (ELISA). Microplate wells (Falcon^®^ 96-well flat-bottom, Corning Life Sciences, Corning, NY, USA) were coated via solid-phase adsorption with 0.1 µg of venom reconstituted in 100 µL of coating buffer (0.1 M Tris, 0.15 M NaCl, pH 9.0) and incubated overnight at room temperature. Nonspecific binding sites were blocked with 100 µL of 1% bovine serum albumin in phosphate-buffered saline (BSA-PBS; 0.04 M phosphates, 0.12 M NaCl, pH 7.2) for 90 min. Serial dilutions of either the antivenom (1:500 to 1:1,093,500) or non-immune equine serum (negative control) were added to the wells and incubated for 90 min at room temperature. Between each incubation step, plates were subjected to five washing cycles using PBS containing 0.05% Tween-20 (PBS-T, pH 7.2). A peroxidase-conjugated anti-horse IgG secondary antibody (1:8000; Sigma-Aldrich) was added and incubated for 90 min. Color development was initiated by adding 100 µL of chromogenic substrate solution (2 mg/mL *o*-phenylenediamine in 0.1 M sodium citrate, pH 5.0, supplemented with 4 µL of 30% H_2_O_2_ per 10 mL of substrate). The reaction was monitored by recording absorbance values at 492 nm using a Multiskan Sky spectrophotometer (Thermo Fisher Scientific, Waltham, MA, USA). All assays were executed in triplicate ( n=3 ), and antibody reactivity was quantified and expressed as raw absorbance values at 492 nm.

To evaluate the antigenic recognition of the INS antivenom against individual venom components, a second ELISA was executed using the major fractions resolved via RP-HPLC. Microplates were coated with 0.1 μg of each fraction per well, and the immunoassay was performed as detailed above utilizing a fixed 1:1000 dilution of the antivenom. Non-immune equine serum was processed in parallel as a negative baseline control. Similarly, the capacity of the antivenom to cross-recognize individual venoms from three distinct *M. ancoralis* specimens was assessed at the same dilution following the same protocol.

Finally, the capacity of the INS anticoral antivenom to neutralize the lethal efficacy of *M. ancoralis* venom was evaluated via a pre-incubation assay. Groups of three Swiss-Webster mice (body weight: 18–20 g) received intraperitoneal (i.p.) injections of a 300 μL volume containing a challenge dose of 2xLD_50_ of venom. This mixture had been previously incubated for 30 min at 37 °C with the antivenom at a fixed ratio of 0.2 mg of venom per mL of antivenom, a proportion selected based on established neutralization models for related elapid venoms [24,25]. Control groups were challenged with an equivalent venom dose pre-incubated with PBS vehicle instead of antivenom. Survival and mortality were recorded over a 48-h observation period.

### 2.7. Molecular Analysis and Phylogenetic Reconstruction

Genomic DNA was isolated from a buccal swab collected from the *M. ancoralis* specimen using the E.Z.N.A.^®^ Tissue DNA Kit (Omega Bio-tek, Norcross, GA, USA; Cat. No. D3396-01), following the manufacturer’s instructions. A fragment of the mitochondrial ND4 gene (approximately 700 bp) was amplified by PCR using the primer pair ND4F (5′-CACCTATGACTACCAAAAGCTCATGTAGAAGC-3′; [26] and LEU (5′-ATTACTTTTACTTGGATTTGCACCA-3′; [27]. Each 25 μL PCR reaction contained 1 μL of genomic DNA (2 ng/μL), 0.2 μM of each primer, 1× PCR reaction buffer, 0.2 mM of each dNTP, 1.5 mM MgCl_2_, 1 U Platinum Taq DNA Polymerase, and nuclease-free water to the final volume. PCR amplification was performed with an initial denaturation at 94 °C for 5 min, followed by 35 cycles of 95 °C for 45 s, 55 °C for 45 s, and 72 °C for 1 min, with a final extension at 72 °C for 10 min. Amplicons were verified by electrophoresis on a 1.5% agarose gel stained with GelRed. Positive controls (previously confirmed ND4-positive DNA) and negative controls, including a no-template control (NTC), were included in every PCR run to monitor amplification success and potential contamination.

PCR products were purified and sequenced by the Sanger method on an ABI 3500 automated capillary sequencer (Applied Biosystems). Sequencing was performed in both forward and reverse directions using the amplification primers. Raw chromatograms were assembled, manually inspected, and edited in Geneious Prime v2026.0.2 [28]). To confirm sequence integrity, ND4 nucleotide sequences were translated into amino acid sequences and screened for frameshifts, premature stop codons, and other potential sequencing artefacts using GeneDoc [29]). The ND4 sequence was deposited in GenBank under accession number PZ569421.

### 2.8. Phylogenetic Reconstruction

To determine the phylogenetic placement of the coral snake *Micrurus ancoralis* from Colombia, a dataset comprising 49 individual sequences representing 46 species of coral snakes was used (one sequence per voucher specimen). Sequences of *Calliophis bivirgatus* and *Micruroides euryxanthus* were included as outgroups. Because ND4 was the marker most consistently represented across available taxa, subsequent analyses were based on this mitochondrial gene. Voucher information, sampling localities, and GenBank accession numbers for all sequences analyzed are provided in Supplementary Table S1.

Protein-coding sequences were aligned in PhyloSuite v1.2.3 [30], using the MACSE algorithm [31] and the vertebrate mitochondrial genetic code. The final alignment comprised 627 bp.

Phylogenetic relationships were inferred under both Maximum Likelihood (ML) and Bayesian frameworks. Maximum Likelihood (ML) analyses were performed in IQ-TREE v2.2.0 implemented in PhyloSuite. The best-fitting nucleotide substitution model was selected using ModelFinder [32] under the Bayesian Information Criterion (BIC), resulting in the GTR + I+ G4 + FO model. Branch support was assessed with 10,000 ultrafast bootstrap replicates. No codon partitioning scheme was applied, and the complete ND4 alignment was analyzed as a single partition.

Bayesian analyses were performed in MrBayes v3.2.7 using the GTR + I+ G substitution model. Although ModelFinder selected GTR + I + G4 + FO for the ML analysis, GTR + I + G was used for Bayesian inference because the +FO component is specific to IQ-TREE and is not implemented in MrBayes. Two independent Markov chain Monte Carlo (MCMC) analyses, each consisting of four chains, were run for 6 × 10^6^ generations with trees sampled every 1000 generations. The first 25% of samples were discarded as burn-in. Convergence was assessed using the average standard deviation of split frequencies (ASDSF = 0.008658), effective sample size (ESS), and potential scale reduction factor (PSRF). The ASDSF was below 0.01, all parameters showed ESS values >300, and PSRF values were approximately 1.0, indicating adequate convergence and mixing of the independent MCMC runs. The resulting phylogenetic hypotheses were visualized and annotated in iTOL [33], whereas final graphical elements were assembled using BioRender.

### 2.9. Statistical Analysis

Experimental datasets derived from quantitative in vitro enzymatic assays ( ${\mathrm{PLA}}_{2}$ and LAAO), in vivo biological evaluations (edema and myotoxicity), and individual venom variation comparisons were expressed as mean ± standard deviation (SD). Normality was evaluated using the Shapiro–Wilk test, and homoscedasticity was confirmed using Brown–Forsythe and Bartlett’s tests prior to parametric analysis. Statistically significant differences among experimental groups were determined via one-way analysis of variance (ANOVA) followed by Tukey’s post-hoc test for multiple pairwise comparisons. Differences were considered statistically significant when p<0.05 . All statistical analyses and graphical plotting were executed using GraphPad Prism software version 8.0 (GraphPad Software, San Diego, CA, USA).

### 2.10. AI-Assisted Figure Generation

The digital illustrations in Figure 1 were assisted by the generative artificial intelligence tool Gemini (Version 3.5 Flas; Google LLC, Mountain View, CA, USA; accessed on 17 June 2026). The tool was utilized to transform an original reference photograph taken by the authors into a vector/schematic format for enhanced visual clarity. The final output was strictly verified by the authors to ensure scientific accuracy and alignment with the original biological specimen.

## 3. Results

### 3.1. Chromatographic, Electrophoretic and Proteomic Profiles

**Figure 2:**
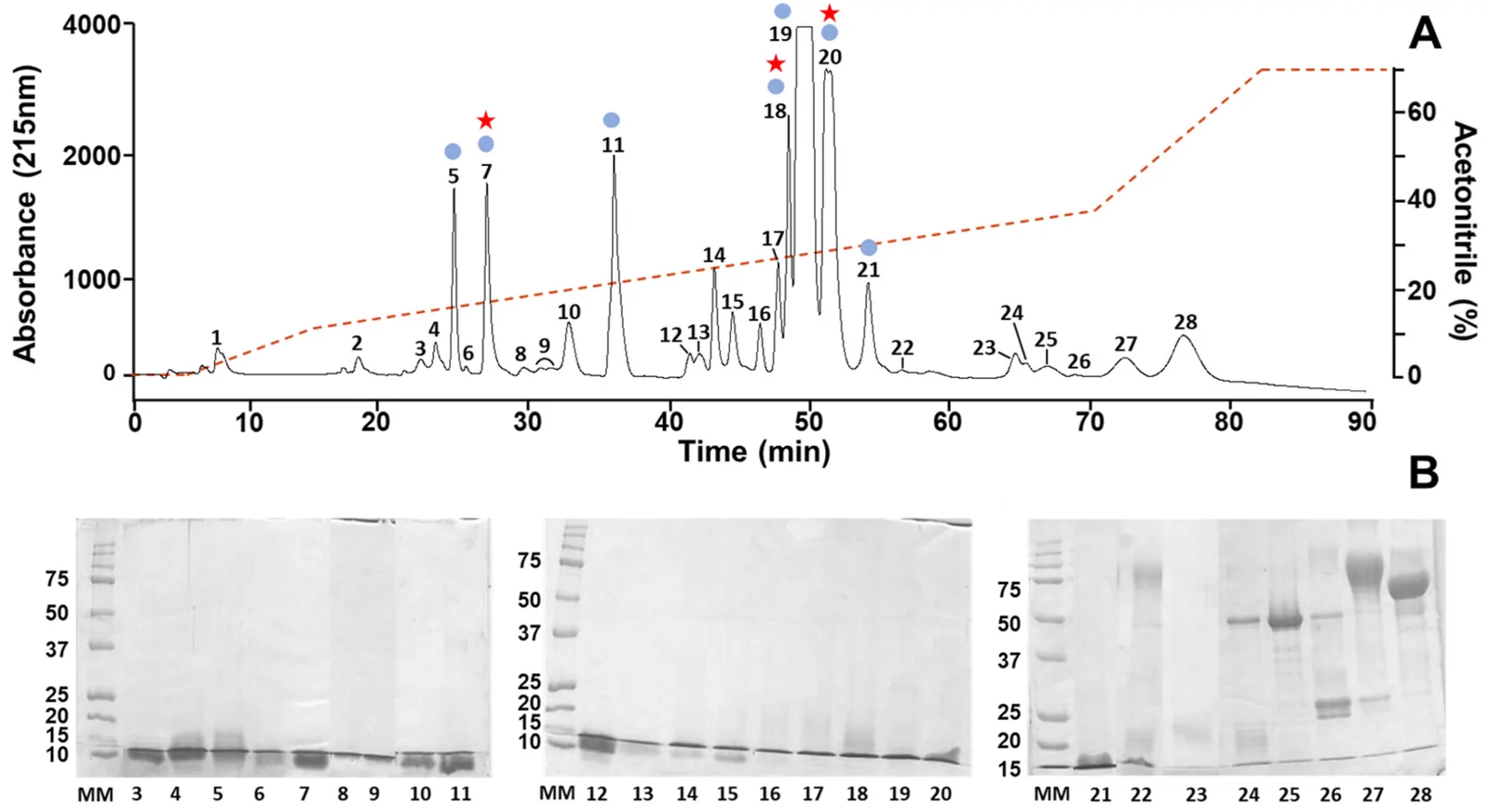
Chromatographic and electrophoretic profiles of *M. ancoralis* venom. (**A**) RP-HPLC profile; Blue dots indicate the fractions evaluated for toxicity, and red stars denote the lethal fractions. The dashed line represents the linear gradient of solvent B (acetonitrile containing 0.1% TFA). (**B**) SDS-PAGE (12%) of each chromatographic fraction, under reducing conditions. Molecular mass markers (in kDa) are shown to the left of samples. Some fractions that were run in independent gels are stitched into the images shown in (**B**).

**Figure 3:**
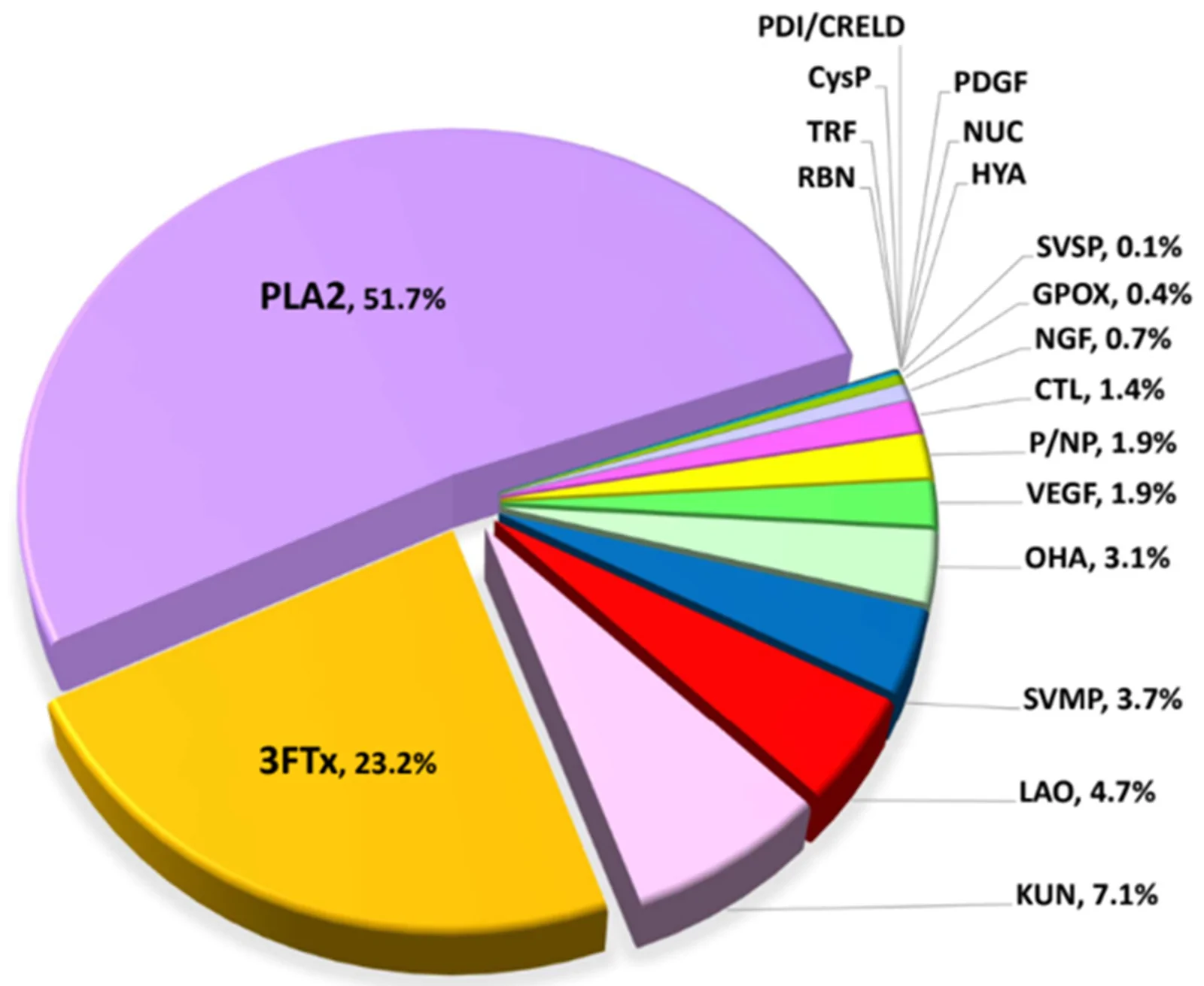
Venom proteomic composition of *M. ancoralis*. The pie chart displays the relative abundance (%) of venom components classified into protein families, expressed as percentages of the total protein content. The profile is dominated by phospholipases A_2_ (PLA_2_) and three-finger toxins (3FTx), followed by Kunitz-type inhibitors (KUN), L-amino acid oxidase (LAO), snake venom metalloproteinases (SVMP), ohanin (OHA), vascular endothelial growth factor (VEGF), peptides/non-proteinaceous components (P/NP), C-type lectin-like (CTL), nerve growth factor (NGF), glutathione peroxidase (GPOX), snake venom serineproteinases (SVSP), hyaluronidase (HYA), nucleotidases (NUC), platelet-derived growth factors (PDGF), ribonucleotidase (RBN), translation-rich factor (TRF), cysteine protease inhibitors (CySP). Full details of protein identifications are provided in Supplementary Table S2.

*M. ancoralis* venom was fractionated by RP-HPLC (Figure 2A), which yielded 28 peaks. These fractions were then submitted to one-dimensional SDS-PAGE (Figure 2B). Low molecular mass proteins (below 15 kDa) were detected in fractions 3 through 21. The proteomic composition, expressed as a percentage of the total venom proteome (Figure 3), was dominated by PLA_2_s, accounting for 51.7% of the total protein content, followed by 3FTxs (23.2%). Other protein families detected at minor proportions included Kunitz-type serine proteinase inhibitors (KUN; 7.1%), L-amino acid oxidases (LAAO; 4.7%), snake venom metalloproteinases (SVMP; 3.7%), ohanin (OHA; 3.1%), vascular endothelial growth factors (VEGF) and peptidic or non-proteinaceous material (P/NP) with 1.9%, and C-type lectin-like proteins (CTL; 1.4%).

Several additional families, such as Glutathione peroxidase (GPOX), hyaluronidase (HYA), nucleotidase (NUC), snake venom serine proteinase (SVSP), phosphodiesterase (PD), ribonucleotidase (RBN), translation-rich factor (TRF), cysteine protease inhibitors (CysP), and platelet-derived growth factor (PDGF), were detected in trace amounts (<0.1%). A small fraction of the proteome (1.9%) remained unidentified.

When the venom was analyzed by using a ‘shotgun’ proteomic strategy, components assigned to 9 protein families were identified: PLA_2_s (10 proteins), 3FTxs (5 proteins), OHA (2 proteins), nerve growth factor (NGF) (1 protein), VEGF (1 protein), SVMP (5 proteins), L-aminoacid oxidase (LAAO) (4 proteins), HYA (1 protein), and SVSP (2 proteins).

### 3.2. Snake Venom Functional Characterization

**Figure 4:**
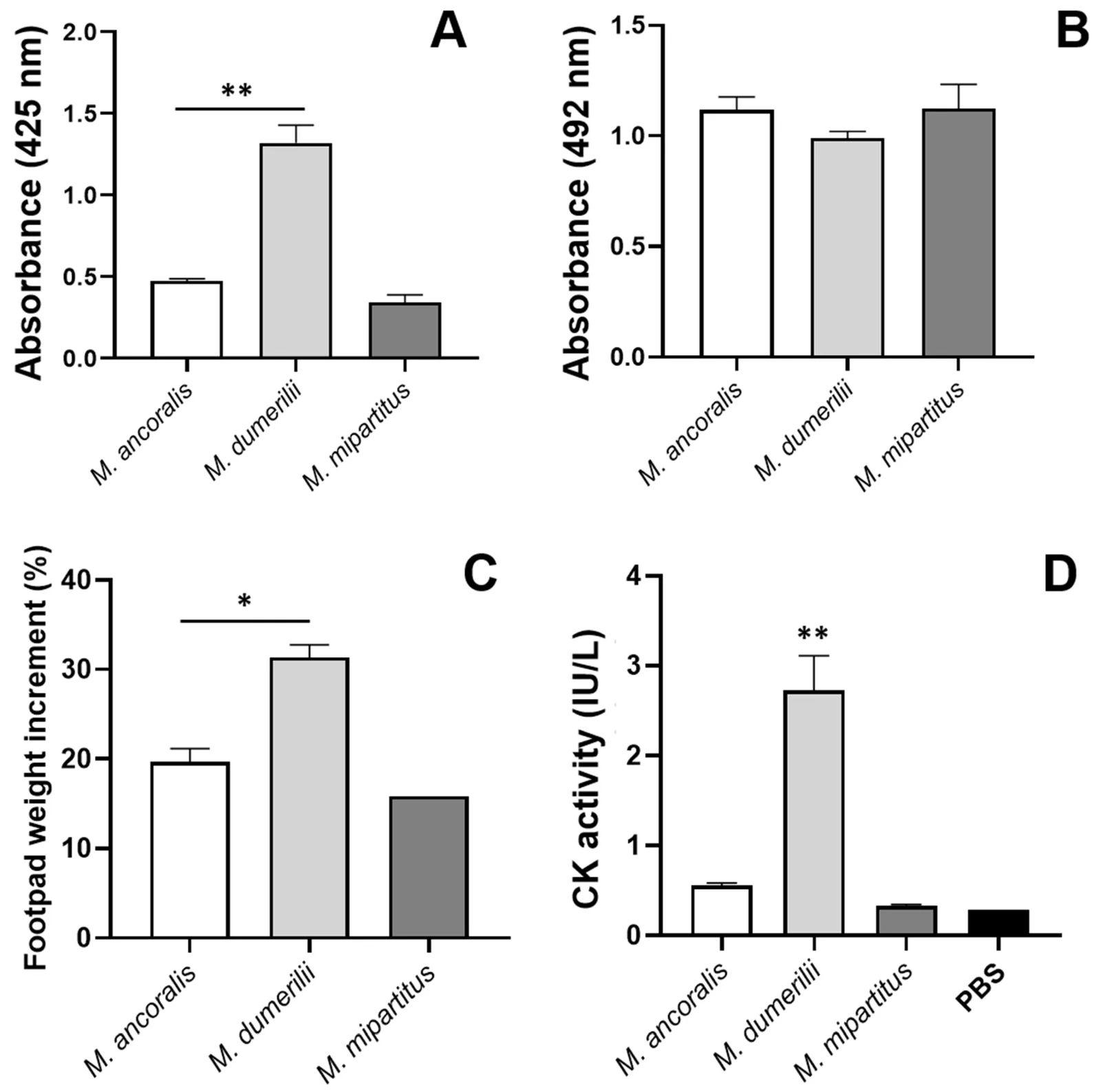
In vitro and in vivo activities of *Micrurus ancoralis* venom. (**A**) Phospholipase A_2_ activity on 4-nitro-3-octanoyloxy-benzoic acid substrate, 20 µg. (**B**) L-amino acid oxidase activity, 20 µg. (**C**) Edema-forming activity in the mouse footpad assay, 5 µg. (**D**) Myotoxic activity in mice, 5 µg intramuscular. PBS was used as a control. Bars represent mean ± SD (*n* = 3). Statistically significant differences between species or compared to the control group are indicated by asterisks (** *p* = 0.001; * *p* < 0.05).

*M. ancoralis* venom was lethal to mice, with an i.p. LD_50_ of 45.2 µg per mouse (2.37 µg/g body weight). Under the experimental conditions tested, the venom did not show detectable proteolytic or amidolytic activity. Its PLA_2_ activity on 4-NOBA was lower in comparison to *M. dumerilii* venom, and alike that of *M. mipartitus* venom (Figure 4A). A similar pattern was observed for both edema-forming and myotoxic activities, where *M. dumerilii* venom showed the highest activities (Figure 4C,D). On the other hand, LAO activity was comparable across all three venoms (Figure 4B).

### 3.3. Characterization of Most Abundant Venom Fractions

Seven chromatographic fractions (F5, F7, F11, F18, F19, F20, F21) were selected on the basis of their higher abundance, and were screened for lethality (i.p.) in mice (Only three of them (F7, F18, and F20) tested positive at the level of 50 µg/mouse (Figure 2). The intact mass analyses showed in fraction F7 monoisotopic molecular masses of 6844.32 Da and 6863.31 Da, consistent with the presence of 3FTxs and the lack of detectable PLA_2_ activity. Fraction F18 displayed PLA_2_ activity and contained components with monoisotopic masses of 13,473.91 Da, 13,490.90 Da, 13,511.89 Da, and 13,547.58 Da. Finally, fraction F20 exhibited PLA_2_, edema-forming, myotoxic, and lethal activities (LD_50_: 45.6 µg/mouse). In this fraction the molecular masses found were 13,392.78 Da, 13,411.79 Da, 13,429.79 Da, 13,454.73 Da, and 13,473.77 Da, all of which are compatible with assignment to PLA_2_.

### 3.4. Immunological Recognition and Neutralization of M. ancoralis Venom by the Therapeutic Equine Anti-Coral Antivenom (INS)

**Figure 5:**
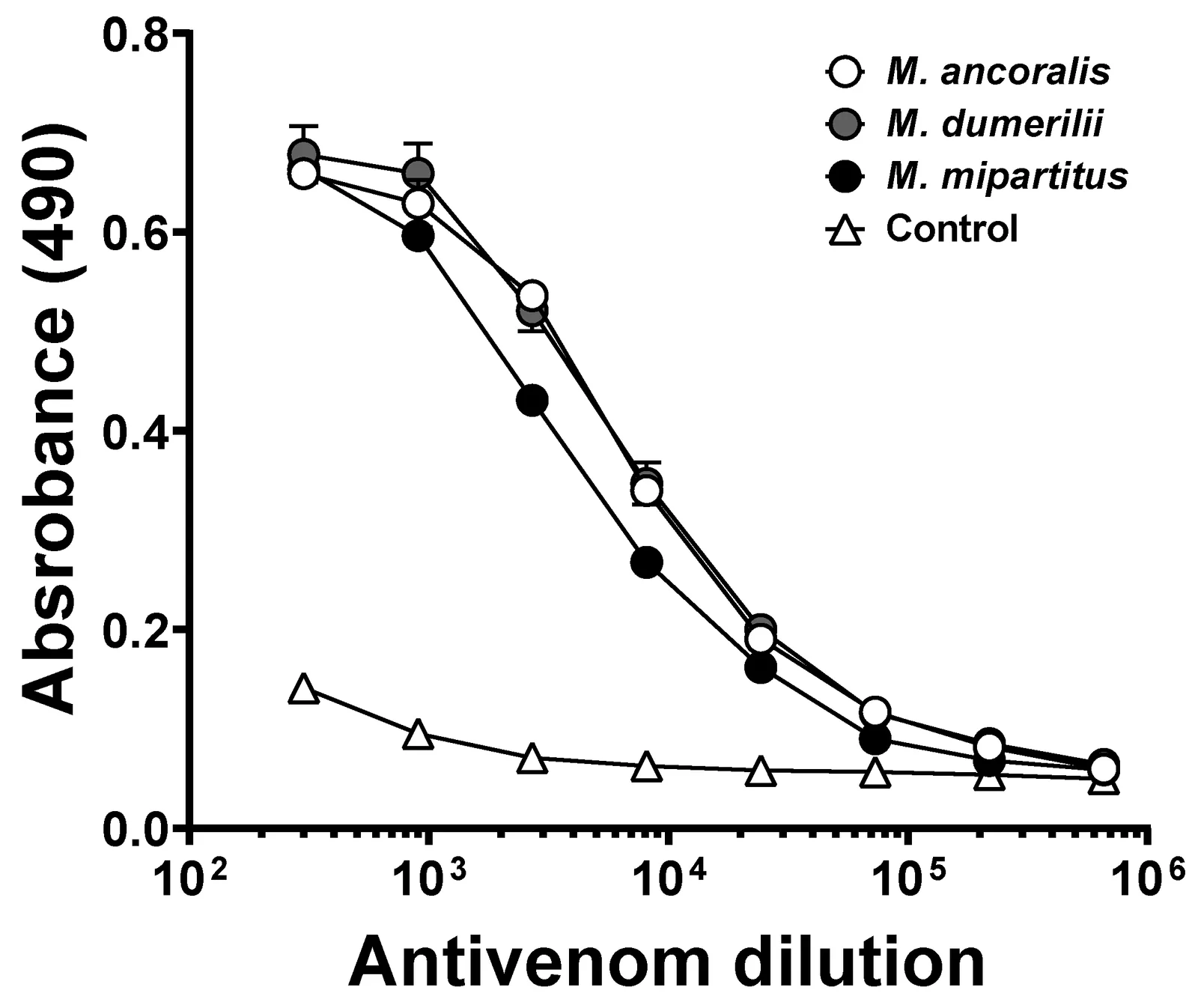
Cross-recognition of *Micrurus ancoralis* venom by a therapeutic equine anti-coral antivenom (INS). Venoms from *Micrurus mipartitus* and *M. dumerilii* were included for comparative analysis of antibody titers. Control: non-immunized horse serum. Each point represents the mean ± standard deviation (SD) of three replicate wells (*n* = 3).

**Figure 6:**
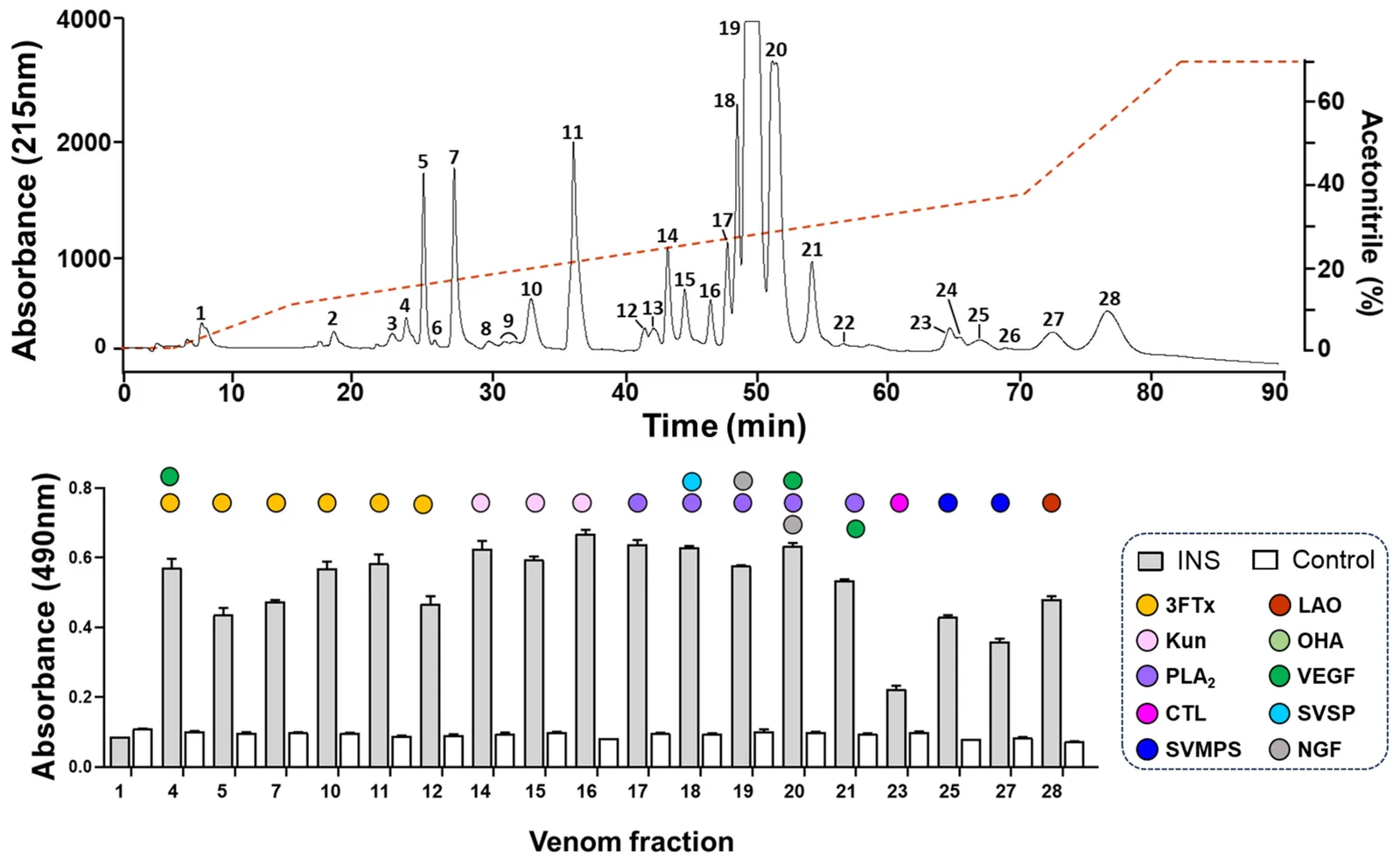
Immunorecognition of the main RP-HPLC fractions of *Micrurus ancoralis* venom by a therapeutic equine anti-coral antivenom (INS). Colored circles indicate the most abundant protein families identified in each chromatographic fraction (proteins with a relative abundance ≥0.1% in each fraction were included, according to Supplementary Table S2). Non-immune equine serum was used as a negative control. The dashed line represents the linear gradient of solvent B (acetonitrile containing 0.1% TFA). Each point represents the mean ± standard deviation (SD) of three replicate wells (*n* = 3).

The INS antivenom exhibited comparable immunorecognition profiles against the whole venoms of *M. ancoralis*, *M. dumerilii*, and *M. mipartitus* in ELISA titrations (Figure 5). Immunorecognition of the most abundant RP-HPLC fractions was also assessed (Figure 6). Fractions enriched in PLA_2_ and Kunitz-type (KUN) proteins displayed the highest signal intensities, whereas fractions containing three-finger toxins (3FTxs) were recognized to a moderate to lower extent. The lowest level of immunorecognition was observed for fractions containing C-type lectins.

Finally, the INS anticoral antivenom effectively neutralized the i.p. lethal activity of 2 × LD_50_ of *M. ancoralis* venom, when both were preincubated at a venom/antivenom ratio of 200 µg venom/mL antivenom, preventing death in all mice.

**Figure 7:**
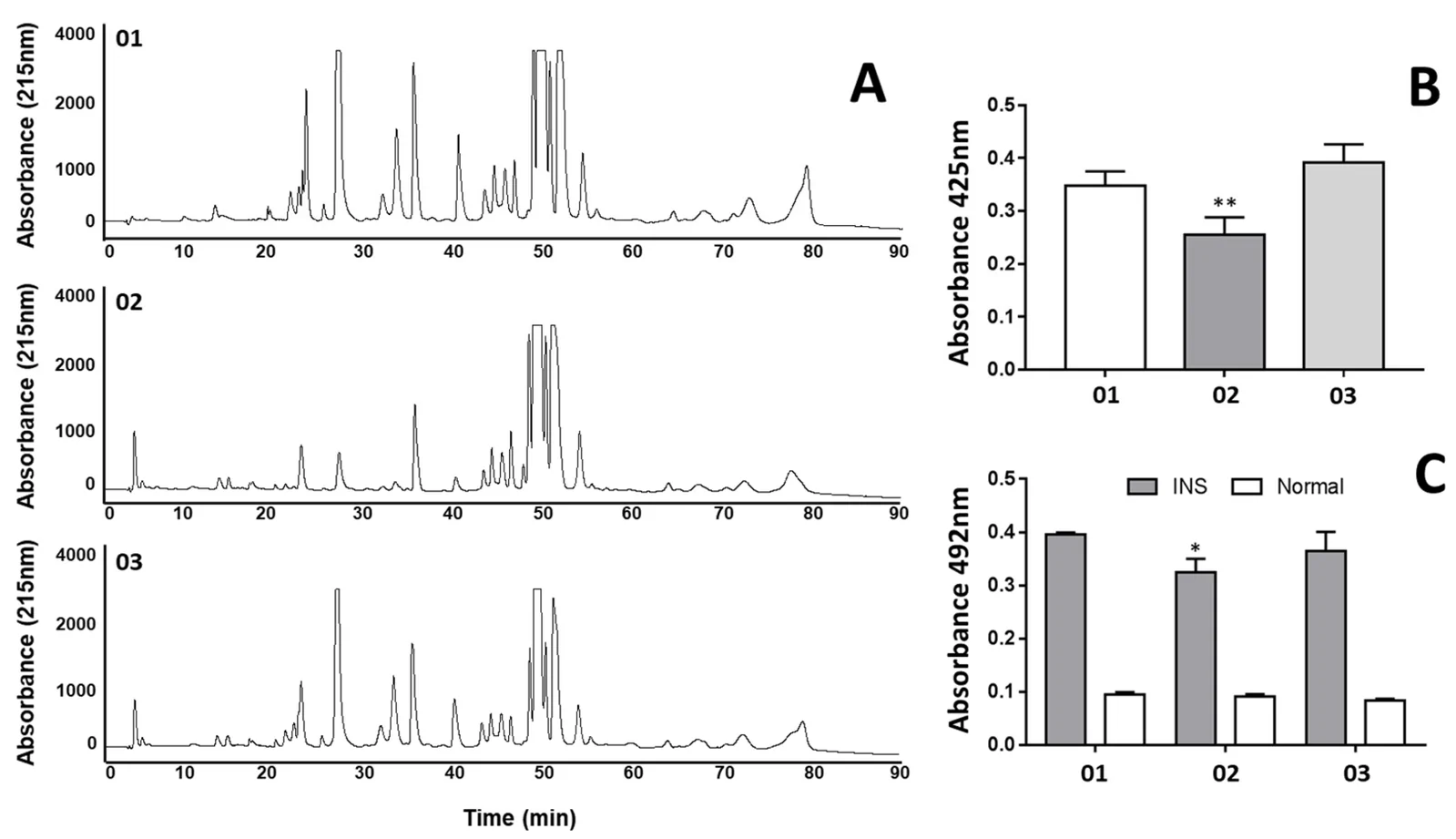
Individual variability in the venom of three *Micrurus ancoralis* specimens. (**A**) RP-HPLC chromatographic profiles of crude venom samples from three individual *M. ancoralis* specimens, illustrating some differences in elution patterns and peak intensities. (**B**) PLA_2_ enzymatic activity (20 µg venom) on 4-nitro-3-(octanoyloxy) benzoic acid substrate. (**C**) Immunorecognition of each venom sample by the INS-coral snake antivenom (1:1000 dilution) in ELISA. Normal: non-immunized rabbit serum. Each bar represents the mean ± standard deviation (SD) of three technical replicates (*n* = 3). Statistically significant differences between specimens are denoted by asterisks (** *p* = 0.001; * *p* < 0.05).

To evaluate intraspecific venom variability and the ability of the INS antivenom to recognize venoms from individual specimens, venoms of three *M. ancoralis* specimens were fractionated by RP-HPLC under the conditions described previously. The individual chromatographic profiles appear qualitatively conserved across the three specimens, although with some variations in the relative intensity of some peaks (Figure 7A). Notably, specimen 02 exhibited reduced abundance in fractions 5, 7, 10, and 11, all of which correspond to 3FTxs (Figure 7A). Further, this specimen showed lowest PLA_2_ activity on 4-NOBA substrate (Figure 7B). In the same way, immunorecognition assays using the INS antivenom revealed similar signal intensities for specimen 01 and 03 venoms, whereas the venom from specimen 02 was recognized to a lesser degree (Figure 7C).

**Figure 8:**
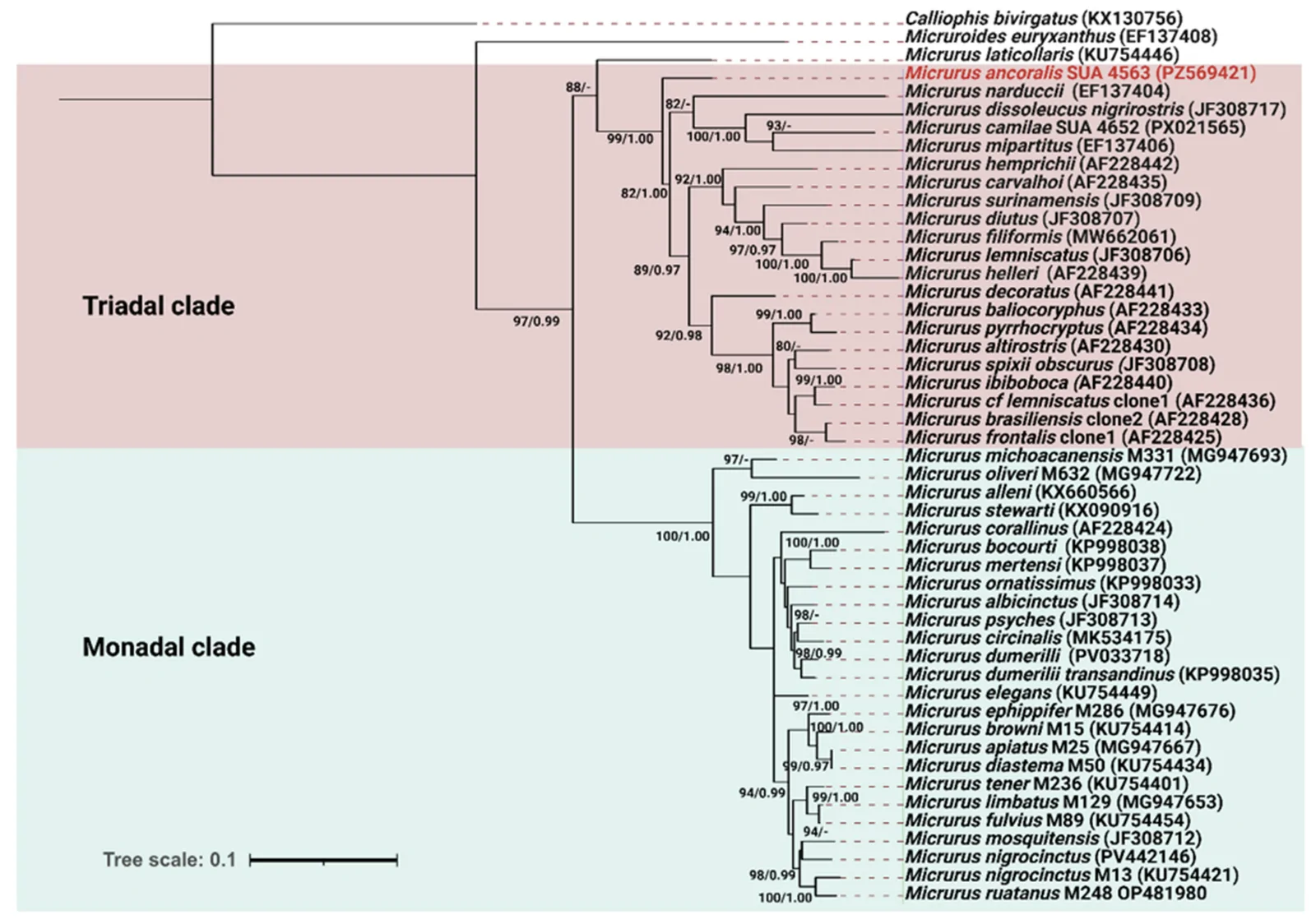
Phylogenetic placement of *Micrurus ancoralis* inferred from mitochondrial ND4 sequences. Maximum Likelihood (IQ-TREE) and Bayesian Inference (MrBayes) phylogenetic analyses were performed independently using the same 627-bp ND4 alignment. Because both analyses recovered congruent topologies, only the Maximum Likelihood tree is shown. Pink shading indicates the triadal clade, and blue shading corresponds to the monadal clade. Nodal support values are presented as UFBoot/PP (e.g., 99/1.00), where the first value corresponds to the Maximum Likelihood ultrafast bootstrap percentage and the second to the Bayesian posterior probability. Nodes lacking sufficient support in the Bayesian analysis are indicated with a dash (“–”). When space constraints prevented placing support values directly at a node, they were positioned above the corresponding branch. *Micrurus ancoralis* (highlighted in red) is the focal taxon of this study, recovered within the Triadal clade with strong support (99/1.00). Outgroup taxa (*Calliophis bivirgatus* and *Micruroides euryxanthus*) were included to root the tree.

The phylogenetic reconstruction based on the mitochondrial ND4 gene recovered the traditional separation between triadal and monadal *Micrurus* clades. Within the triadal clade, *M. ancoralis* clustered with other South American triadal coral snakes, showing a relatively basal position in this lineage. Given that the analysis is based on a single mitochondrial marker, evolutionary interpretations should be considered cautiously (Figure 8).

## 4. Discussion

The New World coral snakes constitute a monophyletic group derived from a single and successful colonization event from the Old World via northern terrestrial land bridges during the Miocene [4]. This radiation comprises two basal evolutionary lineages: *Micruroides* and *Micrurus*. The genus *Micruroides*, represented solely by *M. euryxanthus*, is well-established as the basal sister group to the remaining American radiation clustered within the genus *Micrurus* [1]. To date, the extensive geographic distribution of *Micrurus* across the continent [11] and its exceptional species diversification—currently exceeding 90 recognized species [1]—pose a major taxonomic challenge. This complexity is particularly evident in species delimitation, the resolution of cryptic multi-species complexes, and the fine-scale interpretation of their phylogenetic and evolutionary relationships [4,34,35].

Current molecular systematics supports the division of the genus *Micrurus* into two primary macro-clades: the monadal clade and the triadal/bicolor clade [1,36,37,38]. The monadal clade predominantly groups lineages that diverged early during the colonization of North and Central America, retaining morphological traits considered ancestral within the genus [4]. Conversely, the triadal/bicolor clade embodies the massive evolutionary radiation of the genus in the Neotropics; a highly derived monophyletic block that successfully colonized and diversified across the lowland rainforests and river basins of South America [1].

Despite the remarkable taxonomic richness of *Micrurus* within Colombian territory [2], knowledge regarding local evolutionary relationships remains fragmentary, with only a fraction of these species evaluated under a phylogenetic framework [34,39,40,41,42]. Until the present study, *M. ancoralis* had been entirely excluded from both global and regional molecular datasets. Historically, this species has been assigned to the triadal group based on its diagnostic aposematic coloration pattern, which consists of black rings arranged in triads, bounded by light (white/yellow) rings, and separated by wide red interspaces [10].

In this work, we present a phylogenetic reconstruction inferred from the mitochondrial ND4 gene. Our Maximum Likelihood topology places *M. ancoralis* with strong nodal support within the triadal/bicolor clade, occupying a basal position that bridges the South American triad-bearing species and the bicolor lineages (such as *M. mipartitus* and *M. camilae*). Notably, our phylogenetic inference reveals a direct sister-group relationship with *Micrurus narduccii*, providing the first preliminary molecular evidence suggesting a close evolutionary affinity between these trans-Andean and Amazonian lineages. However, given that this topology relies solely on a single mitochondrial marker, these relationships should be interpreted with caution as tentative hypotheses. Further robust phylogenomic analyses are required to definitively validate these evolutionary links. Nonetheless, these genetic findings are entirely congruent with classic morphological hypotheses, which associate *M. ancoralis* with the “short-tailed” group based on its compact, weakly bilobed hemipenial anatomy [11], as well as traditional lepidosis and color pattern characters [10].

From a venomics perspective, *Micrurus* venoms remain considerably less studied than those of Old World elapids [43,44,45] owing to biological constraints, including their limited venom yield, low survival in captivity, and the difficulty of locating specimens in the field due to their semifossorial habits [46,47]. These limitations have important implications for antivenom production, often necessitating the use of venoms from species with higher venom yields or greater availability, without assurance of adequate immunological coverage against all medically relevant regional species. Envenomings caused by coral snakes are potentially life-threatening due to their high content of neurotoxic venom components, mainly PLA_2_s and 3FTxs [6,7]. These considerations underscore the importance of characterizing the venoms of understudied species such as *M. ancoralis*, and evaluating the corresponding neutralizing ability of antivenoms.

The present work shows that *M. ancoralis* venom is composed predominantly of PLA_2_ enzymes (51.7%) and 3FTxs (23.2%), sharing a similar pattern of PLA_2_ predominance with venoms of other Colombian *Micrurus* species including *M. dumerilii* [48], *M. helleri, M. medemi, M. sangilensis* [49], and *M. camilae* [42]. In contrast, another coral snake of Colombia, *M. mipartitus*, displays a venom with marked predominance of 3FTxs (61%) over PLA_2_s (29%) [50]. The variable venom compositions of *Micrurus* across the Americas have been hypothesized to follow a dichotomous trend, whereby northernmost species tend to exhibit PLA_2_-rich venoms, in contrast to southernmost species, predominantly expressing 3FTxs [51,52]. However, recent studies examining the venoms of an increasing number of *Micrurus* species have described deviations from this apparent geographical trend. For instance, the venom of *M. ephippifer* from Mexico was found to contain 54% of 3FTxs [53]. Elucidating the possible relationships between the variable balance of PLA_2_ and 3FTx proportions and the phylogeographical distribution of *Micrurus* species will require examination of a larger number of coral snakes, as only about one third of their venoms have been analyzed using proteomic approaches. Additionally, intraspecific venom variations within the genus *Micrurus* have been reported in both Central and South American species [54,55,56,57], with changes primarily affecting their protein profiles and functional activities, similar to the subtle differences observed here for Specimen 02 of *M. ancoralis*. Its venom showed the lowest recognition by antivenom. Individual variations had demonstrated changes in antivenom recognition and neutralization capacity, as recently demonstrated for *M. ibiboboca* [58].

An evaluation of enzymatic and toxic activities of *M. ancoralis* venom was performed. Its lethal potency (i.p. LD_50_ = 45.2 µg/mouse) is similar to that reported for *M. camilae* venom (46.7 µg/mouse;) [42], but generally lower than those reported for other Colombian *Micrurus* species, in ascending order of potency: *M. surinamensis* (29.1 µg/mouse;) [59], *M. dumerilii* (23.6 µg/mouse; [48]), *M. helleri* (22.8 µg/mouse; [49]), M. *sangilensis* (15.8 µg/mouse: [49]), *M. mipartitus* (9.0 µg/mouse; [60]), *M. medemi* (8.7 µg/mouse; [49]), and *M. isozonus* (6.3 µg/mouse; [59]). At least three fractions in the venom of *M. ancoralis* were found to induce lethality: F7, assigned to 3FTxs, and F18 and F20, assigned to PLA_2_s. Lethal 3FTxs have previously been isolated from other Colombian *Micrurus* elapids, including mipartoxin-I from *M. mipartitus* (LD_50_ 1.1 µg/mouse [61]) and camilaetoxin from *M. camilae* (LD_50_ 3.2 µg/mouse; [42]). On the other hand, fraction F20 displayed an i.p. LD_50_ of 45.6 µg/mouse, being significantly less potent compared to lethal PLA_2_s previously characterized from the venoms of *M. mipartitus* (1.9 µg/mouse; [50]) and *M. dumerilii* (2.4 µg/mouse; [62]).

*M. ancoralis* venom was shown to induce myonecrosis, as demonstrated by the increase in plasma creatine kinase activity in mice, an effect that has been associated with the action of particular PLA_2_s in *Micrurus* venoms [55,63,64]. This venom also induced a moderate edema in the mouse footpad assay, likely to be attributed to these inflammatogenic enzymes.

In vitro, PLA_2_ activity of *M. ancoralis* venom was confirmed, although being significantly lower compared to *M. dumerilii* venom. On the other hand, no proteolytic activity was detected in *M. ancoralis* venom, at the concentrations tested, probably owing to its low proportion (4.7%) of SVMPs. A similar finding has been reported for the venoms of *M. mipartitus* [50] and *M. dumerilii* [48]. In contrast, species with higher SVMP content, such as *M. helleri, M. medemi*, and *M. sangilensis* (13.1%, 9.6%, and 11.8%, respectively; [49]), and *M. camilae* (9.9%; [42]) exhibited measurable proteolytic activity.

Immunoassays demonstrated the ability of INS anticoral antivenom to cross-recognize *M. ancoralis* venom, which is not included in the immunizing formula used for its production. The ELISA titration showed antibody binding with a signal intensity comparable to that observed with the venom of *M. dumerilii*, which is included in the immunizing formula, therefore indicating a high degree of antigenic similarity of the two venoms. Since immunoassays performed with whole venoms cannot discern which components are responsible for the observed antibody signals, an immunoprofile using chromatographically separated venom fractions was additionally performed. The antivenom displayed a broad recognition of fractions, although with a more robust binding against those containing PLA_2_s than against 3FTx-containing fractions. This trend has been widely documented in immunological studies on *Micrurus* venoms [24,51,55,65,66,67,68] and has been attributed to a lower immunogenicity of the 3FTxs owing to their low molecular masses.

Importantly, from a medical point of view, the INS antivenom not only recognized *M. ancoralis* venom and its fractions but also neutralized its lethal effect in a standard mouse preincubation assay. The observed level of protection, of at least 0.2 mg venom/mL antivenom, predicts that envenomings by *M. ancoralis* could be effectively treated by this antivenom.

## 5. Conclusions

This study provides the first phylogenetic analysis of *M. ancoralis* as well as a proteomic and functional characterization of its venom, contributing novel insights into the diversity and toxic activities of venoms from coral snakes found in Colombia. *M. ancoralis* was recovered with strong nodal support within the triadal/bicolor clade, occupying a basal position that bridges the South American triad-bearing species and the bicolor lineages.

*M. ancoralis* venom is predominantly composed of PLA_2_ enzymes, followed by 3FTxs, a profile consistent with the PLA_2_-rich phenotype described for several other *Micrurus* species. Functionally, this venom exhibits moderate lethal potency in mice, as well as myotoxic and edema-inducing effects. In vitro, this venom lacks appreciable proteolytic and amidolytic activities, while displaying PLA_2_ activity, likely responsible for myotoxicity and edema formation. The identification of at least three lethal fractions, including both PLA_2_s and 3FTxs, highlights the contribution of these toxin families to venom toxicity. In particular, the most abundant fraction (F20), composed of PLA_2_ isoforms, may play a role in the pathological effects of the whole venom being lethal, myotoxic, and inflammatogenic. From an immunological perspective, the INS anticoral antivenom demonstrated effective recognition and neutralization of *M. ancoralis* venom.

## Data Availability

The original contributions presented in this study are included in the article/Supplementary Materials. Further inquiries can be directed to the corresponding authors.

## Supplementary Materials

The following supporting information can be downloaded at https://www.mdpi.com/article/10.3390/tropicalmed11080223/s1, Table S1: Voucher information, sampling localities, and GenBank accession numbers for all analyzed sequences; Table S2: Details of protein identifications by MS/MS.

## References

[1] da Silva N.J., Buononato M.A., Pires M.G., Feitosa D.T., da Silva N.J., Porras L.W., Aird S.D., da Costa Prudente A.L. (2021). Capítulo 4. Serpientes de Coral del Nuevo Mundo: Un Resumen. Avances en la Biología de las Serpientes de Coral.

[2] Uetz P., Hošek J. Virginia. The Reptile Database. c2026. http://www.reptiledatabase.org.

[3] Slowinski J.B., Keogh J.S. (2000). Phylogenetic relationships of elapid snakes based on cytochrome b mtDNA sequences. Mol. Phylogenet. Evol..

[4] Zaher H., Grazziotin F.G., Prudente A.L.C., Quadros A.B.A., Trevine V.C., Lamar W.W., da Silva N.J., Porras L.W., Aird S.D., da Costa Prudente A.L. (2021). Chapter 3: Origin and evolution of elapids and New World coralsnakes. Advances in Coralsnake Biology: With an Emphasis on South America.

[5] Greene S. (2020). Coral snake envenomations in Central and South America. Curr. Trop. Med. Rep..

[6] Bucaretchi F., Capitani E.M., Vieira R.J., Rodrigues C.K., Zannin M., da Silva N.J., Casais-e-Silva L.L., Hyslop S. (2016). Coral snake bites (*Micrurus* spp.) in Brazil: A review of literature reports. Clin. Toxicol..

[7] Bisneto P.F., Alcântara J.A., Mendonça da Silva I., de Almeida Gonçalves Sachett J., Bernarde P.S., Monteiro W.M., Kaefer I.L. (2020). Coral snake bites in Brazilian Amazonia: Perpetrating species, epidemiology and clinical aspects. Toxicon.

[8] Nirthanan S. (2020). Snake three-finger α-neurotoxins and nicotinic acetylcholine receptors: Molecules, mechanisms and medicine. Biochem. Pharmacol..

[9] Pereañez J.A., Chakraborti S. (2023). Snake venom phospholipases A_2_ and their roles in snakebite envenomings. Phospholipases in Physiology and Pathology.

[10] Campbell J.A., Lamar W.W., Brodie E.D. (2004). The Venomous Reptiles of the Western Hemisphere.

[11] Roze J.A. (1996). Coral Snakes of the Americans: Biology, Identification and Venoms.

[12] Rodríguez-Guerra A., Torres-Carvajal O., Pazmiño-Otamendi G., Ayala-Varela F., Salazar-Valenzuela D. (2021). Micrurus ancoralis. Reptiles del Ecuador.

[13] Wallach V., Williams K., Boundy J. (2014). Snakes of the World. A Catalogue of Living and Extinct Species.

[14] Barrera-Ocampo F., Renjifo J.M. (2024). Occurrence of Anchor Coralsnake, *Micrurus ancoralis* (Jan, 1872) (Squamata: Elapidae) confirmed in the Magdalena River Valley of Colombia, with novel citizen science distribution records. Anartia.

[15] Lomonte B., Calvete J.J. (2017). Strategies in ’snake venomics’ aiming at an integrative view of compositional, functional, and immunological characteristics of venoms. J. Venom. Anim. Toxins Incl. Trop. Dis..

[16] Lomonte B., Díaz C., Chaves F., Fernández J., Ruiz M., Salas M., Zavaleta A., Calvete J.J., Sasa M. (2020). Comparative characterization of Viperidae snake venoms from Perú reveals two compositional patterns of phospholipase A_2_ expression. Toxicon X.

[17] Mora-Obando D., Guerrero-Vargas J.A., Prieto-Sánchez R., Beltrán J., Rucavado A., Sasa M., Gutiérrez J.M., Ayerbe S., Lomonte B. (2014). Proteomic and functional profiling of the venom of *Bothrops ayerbei* from Cauca, Colombia, reveals striking interspecific variation with *Bothrops asper* venom. J. Proteom..

[18] Wang W.J., Shih C.H., Huang T.F. (2004). A novel P-I class metalloproteinase with broad substrate-cleaving activity, agkislysin, from *Agkistrodon acutus* venom. Biochem. Biophys. Res. Comm..

[19] Kishimoto M., Takahashi T. (2001). A spectrophotometric microplate assay for L-amino acid oxidase. Anal. Biochem..

[20] Erlanger B.F., Kokowsky N., Cohen W. (1961). The preparation and properties of two new chromogenic substrates of trypsin. Archs. Biochem. Biophys..

[21] Gutiérrez J.M., Calvete J.J., Habib A.G., Harrison R.A., Williams D.J., Warrell D.A. (2017). Snakebite envenoming. Nat. Rev. Dis. Prim..

[22] Yamakawa M., Nozaky M., Hokama Z., Ohsaka A., Hayashi K., Sawai Y. (1976). Fractionation of sakishimahabu (*Trimeresurus elegans*) venom and lethal, hemorrhagic and edema-forming activities of the fractions. Toxins: Animal, Plant and Microbial.

[23] Lomonte B., Fernández J. (2022). Solving the microheterogeneity of *Bothrops asper* myotoxin-II by high-resolution mass spectrometry: Insights into C-terminal region variability in Lys49-phospholipase A_2_ homologs. Toxicon.

[24] Fernández J., Vargas N., Pla D., Sasa M., Rey-Suárez P., Sanz L., Gutiérrez J.M., Calvete J.J., Lomonte B. (2015). Snake venomics of *Micrurus alleni* and *Micrurus mosquitensis* from the Caribbean region of Costa Rica reveals two divergent compositional patterns in New World elapids. Toxicon.

[25] Piedrahita J.D., Cardona-Ruda A., Pereañez J.A., Rey-Suárez P. (2024). In-depth immunorecognition and neutralization analyses of *Micrurus mipartitus* and *M. dumerilii* venoms and toxins by a commercial antivenom. Biochimie.

[26] Arevalo E., Davis S.K., Sites J.W. (1994). Mitochondrial DNA sequence divergence and phylogenetic relationships among eight chromosome races of the *Sceloporus grammicus* complex (Phrynosomatidae) in Central Mexico. Syst. Biol..

[27] Stuart B.L., Parham J.F. (2004). Molecular phylogeny of the critically endangered Indochinese box turtle (*Cuora galbinifrons*). Mol. Phylogenet. Evol..

[28] Kearse M., Moir R., Wilson A., Stones-Havas S., Cheung M., Sturrock S., Buxton S., Cooper A., Markowitz S., Duran C. (2012). Geneious Basic: An integrated and extendable desktop software platform for the organization and analysis of sequence data. Bioinformatics.

[29] Nicholas K.B., Nicholas H.B. (1997). GeneDoc: Analysis and Visualization of Genetic Variation.

[30] Zhang D., Gao F., Jakovlić I., Zou H., Zhang J., Li W.X., Wang G.T. (2020). PhyloSuite: An integrated and scalable desktop platform for streamlined molecular sequence data management and evolutionary phylogenetics studies. Mol. Ecol. Resour..

[31] Ranwez V., Douzery E.J.P., Cambon C., Chantret N., Delsuc F. (2018). MACSE v2: Toolkit for the Alignment of Coding Sequences Accounting for Frameshifts and Stop Codons. Mol. Biol. Evol..

[32] Kalyaanamoorthy S., Minh B.Q., Wong T.K.F., von Haeseler A., Jermiin L.S. (2017). ModelFinder: Fast model selection for accurate phylogenetic estimates. Nat. Methods.

[33] Letunic I., Bork P. (2024). Interactive Tree of Life (iTOL) v6: Recent updates to the phylogenetic tree display and annotation tool. Nucleic Acids Res..

[34] Hurtado-Gómez J.P., Arredondo J.C., Carrillo J.F., Zaher H. (2021). Multilocus phylogeny clarifies relationships and diversity within the *Micrurus lemniscatus* complex (Serpentes: Elapidae). Salamandra.

[35] Zaher H., Grazziotin F., Prudente A.L.C., da Siva N.J. (2016). Origem e Evolução Dos Elapideos e Das Cobras-Corais Do Novo Mundo. Cobras-Corais DO Bras..

[36] Reyes-Velasco J., Adams R.H., Boissinot S., Parkinson C.L., Campbell J.A., Castoe T.A., Smith E.N. (2020). Genome-Wide SNPs Clarify Lineage Diversity Confused by Coloration in Coralsnakes of the *Micrurus diastema* Species Complex (Serpentes: Elapidae). Mol. Phylogenet. Evol..

[37] Nascimento L.R.S., Graboski R., da Silva N.J., Prudente A.L.C. (2024). Integrative taxonomy of *Micrurus ibiboboca* (Merrem, 1820) (Serpentes, Elapidae) reveals three new species of coral snake. Syst. Biodivers..

[38] Bayona-Serrano J.D., Akcali C., Hurtado-Gómez J.P., Angarita-Sierra T., Angarita-Sierra T., Ruiz-Gomez F.J. (2024). Chapter 2. Lineages, venom variability, and warning signals in New World Coral snakes. Bites, Venoms, and Venomous Snakes of Colombia.

[39] Renjifo C., Smith E.N., Hodgson W.C., Renjifo J.M., Sanchez A., Acosta R., Maldonado J.H., Riveros A. (2012). Neuromuscular activity of the venoms of the Colombian coral snakes *Micrurus dissoleucus* and *Micrurus mipartitus*: An evolutionary perspective. Toxicon.

[40] Rey-Suárez P., Rojo L.P., Gómez-Robles J., Parra-Moreno S., Pachon-Camelo E., Fuentes-Florez Y., Lomonte B., Fernández J., Sasa M., Núñez V. (2025). *Micrurus nigrocinctus* in Colombia: Integrating Venomics Research, Citizen Science, and Community Empowerment. Toxins.

[41] Rey-Suárez P., Gómez-Robles J., Fernández J., Lomonte B., Sasa M., Saldarriaga-Cordoba M., Pereañez J.A., Aguilera O., Núñez-Rangel V. (2025). Assessment of venom variation and phylogenetic relationships of *Micrurus dumerilii* from three different regions of Colombia. Biochimie.

[42] Gómez-Robles J., Rey-Suárez P., Fernández J., Saldarriaga-Córdoba M., Sasa M., Lomonte B., Núñez V. (2026). First characterization of the venom of the endemic coral snake *Micrurus camilae* (Serpentes: Elapidae) from Colombia: Proteome, toxic activities, immunorecognition, and neutralization by antivenoms. PLoS Negl. Trop. Dis..

[43] Calvete J.J., Lomonte B., Saviola A.J., Bonilla F., Sasa M., Williams D.J., Undheim E.A.B., Sunagar K., Jackson T.N.W. (2021). Mutual enlightment: A toolbox of concepts and methods for integrating evolutionary and clinical toxinology via snake venomics and the contextual stance. Toxicon X.

[44] Lomonte B., Calvete J.J., Fernández J., Pla D., Rey-Suárez P., Sanz L., Gutiérrez J.M., Sasa M., da Silva N.J., Porras L.W., Aird S.D., Prudente A.L. (2021). Venomic analyses of coralsnakes. The Biology of the Coralsnakes.

[45] Tasoulis T., Isbister G.K. (2023). A current perspective on snake venom composition and constituent protein families. Archs. Toxicol..

[46] Henao A., Núñez V. (2016). Maintenance of red-tail coral snake (*Micrurus mipartitus*) in captivity and evaluation of individual venom variability. Acta Biol. Colomb..

[47] Neri-Castro E., Zarzosa V., Benard-Valle M., Rodríguez-Solís A.M., Hernández-Orihuela L., Ortiz-Medina J.A., Alagón A. (2024). Quantifying venom production: A study on *Micrurus* snakes in Mexico. Toxicon.

[48] Rey-Suárez P., Núñez V., Fernández J., Lomonte B. (2016). Integrative characterization of the venom of the coral snake *Micrurus dumerilii* (Elapidae) from Colombia: Proteome, toxicity, and cross-neutralization by antivenom. J. Proteom..

[49] Rodríguez-Vargas A., Franco-Vásquez A.M., Bolívar-Barbosa J.A., Vega N., Reyes-Montaño E., Arreguín-Espinosa R., Carbajal-Saucedo A., Angarita-Sierra T., Ruiz-Gómez F. (2023). Unveiling the venom composition of the Colombian coral snakes *Micrurus helleri*, *M. medemi*, and *M. sangilensis*. Toxins.

[50] Rey-Suárez P., Núñez V., Gutiérrez J.M., Lomonte B. (2011). Proteomic and biological characterization of the venom of the redtail coral snake, *Micrurus mipartitus* (Elapidae), from Colombia and Costa Rica. J. Proteom..

[51] Lomonte B., Rey-Suárez P., Fernández J., Sasa M., Pla D., Vargas N., Bénard-Valle M., Sanz L., Corrêa-Netto C., Núñez V. (2016). Venoms of *Micrurus* coral snakes: Evolutionary trends in compositional patterns emerging from proteomic analyses. Toxicon.

[52] Sanz L., Quesada-Bernat S., Ramos T., Casais-e-Silva L.L., Corrêa-Netto C., Silva-Haad J.J., Sasa M., Lomonte B., Calvete J.J. (2019). New insights into the phylogeographic distribution of the 3FTx/PLA_2_ venom dichotomy across genus *Micrurus* in South America. J. Proteom..

[53] Zarzosa V., Neri-Castro E., Lomonte B., Fernández J., Rodríguez-Barrera G., Rodríguez-López B., Rodríguez-Solís A.M., Olvera-Rodríguez A., Bénard-Valle M., Saviola A. (2025). Integrative transcriptomic, proteomic, biochemical and neutralization studies on the venom of *Micrurus ephippifer*. J. Proteom..

[54] Aird S.D., da Silva N.J. (1991). Comparative enzymatic composition of Brazilian coral snake (*Micrurus*) venoms. Comp. Biochem. Physiol. B.

[55] Fernández J., Alape-Girón A., Angulo Y., Sanz L., Gutiérrez J.M., Calvete J.J., Lomonte B. (2011). Venomic and antivenomic analyses of the Central American coral snake, *Micrurus nigrocinctus* (Elapidae). J. Proteome Res..

[56] Tan N.H., Ponnudurai G. (1992). The biological properties of venoms of some American coral snakes (*Genus micrurus*). Comp. Biochem. Physiol. B.

[57] de Roodt A.R., Lago N.R., Stock R.P. (2012). Myotoxicity and nephrotoxicity by *Micrurus* venoms in experimental envenomation. Toxicon.

[58] Casais-E-Silva L., Vaccas T., Leite L.G.D.S., da Silva L.M.P., Rossini B.C., Ferreira Andrade D., de Fátima Domingues Furtado M., Biondi I., Dos Santos L.D. (2026). Inter and intraspecific variation in Micrurus venoms from Bahia, Brazil and challenges for antivenom efficacy. Biochimie.

[59] Castillo-Beltrán M.C., Hurtado-Gómez J.P., Corredor-Espinel V., Ruiz-Gómez F.J. (2019). A polyvalent coral snake antivenom with broad neutralization capacity. PLoS Negl. Trop. Dis..

[60] Otero R., Osorio R.G., Valderrama R., Giraldo A.C. (1992). Effectos farmacológicos y enzimáticos de los venenos de serpientes de Antioquia y Choco (Colombia). Toxicon.

[61] Rey-Suárez P., Floriano R.S., Rostelato-Ferreira S., Saldarriaga-Córdoba M., Núñez V., Rodrigues-Simioni L., Lomonte B. (2012). Mipartoxin-I, a novel three-finger toxin, is the major neurotoxic component in the venom of the redtail coral snake *Micrurus mipartitus* (Elapidae). Toxicon.

[62] Gómez-Robles J., Rey-Suárez P., Pereañez J.A., Lomonte B., Núñez V. (2023). Antibodies against a single fraction of *Micrurus dumerilii* venom neutralize the lethal effect of whole venom. Toxicol. Lett..

[63] Casais-e-Silva L.L., Texeira C.F.P., Lebrun I., Lomonte B., Alape-Girón A., Gutiérrez J.M. (2016). Lemnitoxin, the major component of *Micrurus lemniscatus* coral snake venom, is a myotoxic and proinflammatory phospholipase A_2_. Toxicol. Lett..

[64] Rey-Suárez P., Núñez V., Saldarriaga-Córdoba M., Lomonte B. (2017). Primary structures and partial toxicological characterization of two phospholipases A_2_ from *Micrurus mipartitus* and *Micrurus dumerilii* coral snake venoms. Biochimie.

[65] Lippa E., Török F., Gómez A., Corrales G., Chacón D., Sasa M., Gutiérrez J.M., Lomonte B., Fernández J. (2019). First look into the venom of Roatan Island’s critically endangered coral snake *Micrurus ruatanus*: Proteomic characterization, toxicity, immunorecognition and neutralization by an antivenom. J. Proteom..

[66] Mena G., Chaves-Araya S., Chacón J., Török E., Török F., Bonilla F., Sasa M., Gutiérrez J.M., Lomonte B., Fernández J. (2022). Proteomic and toxicological analysis of the venom of *Micrurus yatesi* and its neutralization by an antivenom. Toxicon X.

[67] Cardona-Ruda A., Rey-Suárez P., Núñez V. (2022). Anti-Neurotoxins from *Micrurus mipartitus* in the Development of Coral Snake Antivenoms. Toxins.

[68] Rodríguez-Vargas A., Franco-Vásquez A.M., Triana-Cerón M., Alam-Rojas S.N., Escobar-Wilches D.C., Corzo G., Lazcano-Pérez F., Arreguín-Espinosa R., Ruiz-Gómez F. (2024). Immunological cross-reactivity and preclinical assessment of a Colombian anticoral antivenom against the venoms of three *Micrurus* species. Toxins.

